# Improving Lowland Rice (*O. sativa* L. cv. MR219) Plant Growth Variables, Nutrients Uptake, and Nutrients Recovery Using Crude Humic Substances

**DOI:** 10.1155/2015/906094

**Published:** 2015-04-21

**Authors:** Perumal Palanivell, Osumanu Haruna Ahmed, Nik Muhamad Ab Majid, Mohamadu Boyie Jalloh, Kasim Susilawati

**Affiliations:** ^1^Department of Crop Science, Faculty of Agriculture and Food Sciences, Universiti Putra Malaysia Bintulu Sarawak Campus, 97008 Bintulu, Sarawak, Malaysia; ^2^Agriculture and Environment, Borneo Eco-Science Research Center, Faculty of Agriculture and Food Sciences, Universiti Putra Malaysia Bintulu Sarawak Campus, 97008 Bintulu, Sarawak, Malaysia; ^3^Institute of Tropical Forestry and Forest Products (INTROP), Universiti Putra Malaysia, 43400 Serdang, Selangor, Malaysia; ^4^Faculty of Sustainable Agriculture, Universiti Malaysia Sabah Kampus Sandakan, Locked Bag No. 3, 90509 Sandakan, Sabah, Malaysia

## Abstract

High cation exchange capacity and organic matter content of crude humic substances from compost could be exploited to reduce ammonia loss from urea and to as well improve rice growth and soil chemical properties for efficient nutrients utilization in lowland rice cultivation. Close-dynamic air flow system was used to determine the effects of crude humic substances on ammonia volatilization. A pot experiment was conducted to determine the effects of crude humic substances on rice plant growth, nutrients uptake, nutrients recovery, and soil chemical properties using an acid soil mixed with three rates of crude humic substances (20, 40, and 60 g pot^−1^). Standard procedures were used to evaluate rice plant dry matter production, nutrients uptake, nutrients recovery, and soil chemical properties. Application of crude humic substances increased ammonia volatilization. However, the lowest rate of crude humic substances (20 g pot^−1^) significantly improved total dry matter, nutrients uptake, nutrients recovery, and soil nutrients availability compared with crude humic substances (40 and 60 g pot^−1^) and the normal fertilization. Apart from improving growth of rice plants, crude humic substances can be used to ameliorate acid soils in rice cultivation. The findings of this study are being validated in our ongoing field trials.

## 1. Introduction

Ultisols and Oxisols are the most common agricultural soils in the tropics. Besides being highly weathered, these soils are acidic, very low in cation exchange capacity (CEC), and inherently low in nutrients [1]. The large amounts of oxides and hydroxides of Fe and Al in Ultisols and Oxisols have been associated with the abundance of variable charge colloids, low pH, and low CEC [2, 3]. These oxides and hydroxides commonly fix large amounts of soluble phosphate leading to low concentrations of available Phosphorus (P) in soil solution [4, 5]. Furthermore, in lowland rice fields (waterlogged condition) urea is mostly lost through NH_3_ volatilization. Ammonia volatilization is higher in waterlogged condition compared to nonwaterlogged condition [6, 7] and this NH_3_ volatilization occurs when urea is hydrolyzed to ammonium carbonate (equation (1)). Subsequently, ammonium carbonate decomposes into CO_2_, H_2_O, and NH_3_ (equations (2) and (3)) [8].

*Urea Hydrolysis*

(1) $$\begin{array}{c} {\left(\text{N}{\text{H}}_{2}\right)}_{2}\text{CO}+2{\text{H}}_{2}\text{O}\overset{\text{Soil urease}}{\to }{\left(\text{N}{\text{H}}_{4}\right)}_{2}{\text{CO}}_{3} \end{array}$$

(2) $$\begin{array}{c} {\left(\text{N}{\text{H}}_{4}\right)}_{2}{\text{CO}}_{3}+2{\text{H}}^{+}⟶2\text{N}{\text{H}}_{4}^{+}+\text{C}{\text{O}}_{2}+{\text{H}}_{2}\text{O} \end{array}$$

(3) $$\begin{array}{c} \text{N}{\text{H}}_{4}^{+}+\text{O}{\text{H}}^{-}⇌\text{N}{\text{H}}_{3}+{\text{H}}_{2}\text{O}\;\left(\text{see}\;\left[8\right]\right) \end{array}$$

Thus the aforementioned problems lead to nutrients unavailability for rice plant growth and development. To overcome the stated problems, farmers apply fertilizers excessively to attain higher yield. However, unbalanced use of N and P fertilizers causes environmental problems such as eutrophication, groundwater pollution, acid rain deposition, soil acidification, and greenhouse gas emissions [9–12].

Utilization of humic substances in acidic soils was reported not only to have reduced Al^3+^ and Fe^2+^ but also to have increased P [13]. Reduction of Al^3+^ and Fe^2+^ ions in soil solution is essential as this prevents P from being precipitated as strengite, vivianite, variscite, and various minerals of the plumbogummite group [14]. Moreover, P fixation in acid soils can be reduced by increasing soil pH as availability of P varies with variation in soil pH. For example, most of P is available for plant uptake at neutral pH [8]. This suggests that the sawdust ash used to extract crude humic substances in this study can be exploited to increase pH of acidic soils because of their basic nature (pH 11). Crude humic substances which are produced from rice straw compost and sawdust ash can also be used to improve soil chemical properties because these organic substances can increase soil pH and organic matter [15]. Additionally, the high CEC of rice straw compost can be exploited to improve CEC of acid soils and to as well retain cations such as NH_4_^+^, K^+^, Ca^2+^, and Mg^2+^. In addition, good retention of cations including NH_4_^+^ ion, ammonia volatilization from urea, and nutrients leaching could be significantly reduced. Ammonia volatilization from urea as an example can be reduced with application of materials which are high in CEC [16–18]. Humic substances which are high in CEC have been used to control ammonia volatilization in nonwaterlogged condition. In nonwaterlogged conditions, humic acids and fulvic acids from tropical peat, coal, palm oil mill effluent sludge, and crude humins from composts [19–23] have been used to reduce NH_3_ loss from urea. In waterlogged soils, Clinoptilolite zeolite has been used to reduce ammonia volatilization [17, 18, 24].

Apart from fewer studies which had been carried out to determine the effects of humic substances on ammonia loss and soil chemical properties of waterlogged soil, isolation (extraction, fractionation, and purification) of humic acids, fulvic acids, and humins is laborious and expensive. Hence, in this present study, rice straw compost was rather mixed with sawdust ash to produce crude humic substances. We hypothesized that the application of crude humic substances which are high in CEC could reduce NH_3_ volatilization from urea, increase rice plant growth, nutrients uptake, recovery, and as well improving soil chemical properties in waterlogged condition. Thus, this study was conducted to determine the effects of mixing an acid soil (Typic Paleudults) with crude humic substances at three rates (20, 40, and 60 g pot^−1^) in waterlogged condition on NH_3_ volatilization from urea, rice plant growth, nutrients uptake, and recovery and improve soil chemical properties.

## 2. Materials and Methods

### 2.1. Soil Sampling, Preparation, and Characterization

Typic Paleudults (Bekenu Series) soil was sampled at 0 to 25 cm in an uncultivated area of Universiti Putra Malaysia Bintulu Sarawak Campus, Malaysia (latitude 3°12′N and longitude 113°4′E). The soil was air-dried, ground, and sieved to pass to a 5 mm sieve. The soil was analyzed before and after the pot study for pH in distilled water (at ratio of 1 : 2.5 soil : water) using a digital pH meter [25], total carbon using loss-on-ignition method [26], total organic carbon (TOC) and organic matter (OM) content using Walkley-Black method [27], cation exchange capacity (CEC) using the ammonium acetate leaching method [28], total N using Kjeldahl method [29], and available NO_3_^−^ and exchangeable NH_4_^+^ [30]. Exchangeable cations and available P were extracted using the Double Acid Method [31], after which cations were determined using Atomic Absorption Spectrometer (AAnalyst 800, Perkin Elmer Instruments, Norwalk, CT), whereas available P was determined using the Blue Method [32]. Total titratable acidity was determined using acid-base titration method [33]. The selected chemical and physical properties of the soil (Table 1) used in this study are typical of Typic Paleudults (Bekenu series) and they are consistent with those reported by Paramananthan [34] except for CEC, exchangeable calcium, and magnesium.

### 2.2. Rice Straw Compost and Sawdust Ash Production

The compost used in this study is produced from mixture of rice straw (75%) + chicken dung (15%) + molasses (6%) + urea (2%) + rock phosphate (2%) [35]. Sawdust dust was incinerated at 600°C using muffle furnace for 10 hours in an aluminium ware (size 30 cm length × 17 cm width × 8 cm height) until almost white ash is produced.

### 2.3. Rice Straw Compost and Sawdust Ash Characterization

The rice straw compost and sawdust ash were analyzed for pH in distilled water (at ratio of 1 : 10 compost : water; 1 : 100 ash : water) using a digital pH meter [25], total N using Kjedahl method [29], CEC using the ammonium acetate leaching method [28], organic matter, and total organic carbon content using loss-on ignition method [36]. Total P and cation contents in the rice straw compost and sawdust ash were extracted using the single dry ashing method [28]. Phosphorus in the extract was quantified using the Blue Method [32], whereas cations content were determined using atomic absorption spectrophotometer (AAnalyst 800, Perkin Elmer Instruments, Norwalk, CT). The molarity of sawdust ash (at ratio of 1 : 100 ash : water) was determined using acid-base titration [37] with modification of 180 rpm instead of 150 rpm. Humic acids and crude humins content in rice straw compost were determined using method of Ahmed et al. [38] and Palanivell et al. [35, 39]. Total organic carbon, organic matter content, humic acids, and CEC of the rice straw compost and pH and total calcium content of the sawdust ash were higher as expected (Table 2).

### 2.4. Treatments Evaluation for Ammonia Loss from Urea

The ammonia loss incubation study was conducted using close-dynamic air flow system [40, 41]. Treatments were arranged in a Completely Randomized Design (CRD) with three replications. The treatments per 250 g of soil evaluated in 500 mL conical flask were as follows.

- C1: soil alone.
- C2: soil + complete fertilization.
- CHS1: soil + complete fertilization + 20 g rice straw compost + 2 g sawdust ash.
- CHS2: soil + complete fertilization + 40 g rice straw compost + 4 g sawdust ash.
- CHS3: soil + complete fertilization + 60 g rice straw compost + 6 g sawdust ash.

The complete fertilization is equivalent to 1.31 g urea + 1.39 g ERP + 0.88 g MOP + 0.16 g Kieserite + 0.53 g chelated ZnCoBor per experimental unit. The amounts of fertilizers used were scaled down for plant density of 3 rice plants hill^−1^. This fertilizer rate (151 kg ha^−1^ N, 97.8 kg ha^−1^ P_2_O_5_, 130 kg ha^−1^ K_2_O, and 7.6 kg ha^−1^ MgO) was based on the recommended fertilizer for rice by Muda Agricultural Development Authority, Malaysia [42], with additional micronutrients (2.3 kg ha^−1^ B, 4 kg ha^−1^ Cu, and 4 kg ha^−1^ Zn) [43]. The amounts of rice straw compost used in this study were deduced from the literature [44–49], where 5, 10, and 15 tons ha^−1^ equivalent to 20, 40, and 60 g pot^−1^ were used.

The incubation study was carried out by mixing soil with rice straw compost and sawdust ash for the treatments with crude humic substances alone after which the mixture was moistened to 100% of field capacity and left overnight to equilibrate. Before the fertilizers were applied, the water level in each conical flask was maintained at 3 cm from the soil surface to ensure that the system was waterlogged. The water level was marked on the conical flasks. The water level in the conical flask was maintained throughout incubation period (33 days) by adding distilled water as the deficit of the original water level. The fertilizers were applied on the soil surface, and air was passed through the volatilization system at a rate of 2.5 L min^−1^ and volatilized ammonia from urea was captured in 75 mL of 2% boric acid solution with bromocresol green and methyl red indicator. The rate of air flow was measured using a Gilmont flow meter (Gilmont Instrument, Great Neck, NY, USA). The boric acid solution was replaced every 24 h and back titrated with 0.05 M HCl to determine ammonia loss from urea. This measurement was continued until the ammonia loss decreased to 1% of the N added in the system [24, 40].

### 2.5. Germination Media Selection

Before the pot study, germination media selection was done using rice seeds variety of MR219 (commonly used rice seed in Malaysia). This study was carried out using plastic ware measuring 17 cm (length) × 12 cm (width) × 6.5 cm (height) filled with 100 g of germination media and the media were moistened up to 100% of its field capacity. The river sand used in this study was washed with tap water after which it was washed thoroughly with distilled water until the pH of the sand was neutral (pH 7). Ten rice seeds were seeded on each germination medium. The experimental design was Completely Randomized Design (CRD) with 3 replications. The treatments evaluated were as follows.

- G1: 100% sand
- G2: 5% rice straw compost + 95% sand
- G3: 10% rice straw compost + 90% sand
- G4: 15% rice straw compost + 85% sand

After 7 days of seeding, germination rate, root elongation, and shoot elongation were measured using a digital vernier caliper whereas relative seed germination, germination index, relative root elongation, and relative shoot elongation of rice seedling were determined as described by Araújo and Monteiro [50]. The ammonia volatilization and germination medium selection studies were conducted in a laboratory at the Research Center of Universiti Putra Malaysia Bintulu Sarawak Campus, Malaysia, which had an average temperature of 29.6°C (range 26.8–31.9°C) and an average relative humidity of 71.0% (range 50.1–85.1%).

### 2.6. Pot Study

The quantity of the soil used in the pot study was calculated based on the soil's bulk density. A 1 kg of air-dried soil was filled in a pot measuring 12.5 cm (top diameter) × 10 cm (bottom diameter) × 9 cm (height). Before planting, rice seeds variety of MR219 were germinated in a plastic ware filled with germination medium (100% sand) which was selected from the germination medium selection study. The treatments evaluated in this pot study were the same as those used in the ammonia volatilization study. However, fertilization was done as summarized in Table 3.

The pots were filled with soil only in C1 and C2. For CHS1, CHS2, and CHS3, soil was mixed thoroughly with different rates of crude humic substances (20, 40, and 60 g pot^−1^) according to the treatments. Crude humic substances were prepared by mixing sawdust ash, rice straw compost, and water thoroughly (at ratio of 1 : 10 : 100) and left for 24 h before mixing with soil. Potting was done 24 h before transplanting. The water level in each pot was maintained at 2 cm from the soil surface. At 7 days after seeding, rice seedlings were transferred into pots (planting density of 3 seedlings per pot which is equivalent to 3 seedlings hill^−1^) [51]. This pot trial was carried out in a rain shelter at Universiti Putra Malaysia Bintulu Sarawak Campus, Malaysia, and the treatments were arranged in Completely Randomized Design (CRD) with 4 replications. The experimental area has total precipitation of 753 mm, average relative humidity of 85% (range 70–95%), and mean minimum and maximum temperatures of 23.3°C and 32.7°C, respectively, throughout the study period [52].

Plant height, number of tillers, and number of leaves were recorded at harvest (90 days after seeding). The rice plants were harvested and partitioned into aboveground biomass and root before heading stage (end of vegetative stage). This was due to the insufficient amount of soil to support rice plant up to reproduction stage. Besides, it is not economically practical to estimate yield of rice based on pot experiment. The plant parts were oven-dried at 60°C until a constant weight obtained. The oven-dried plant samples were ground using Retsch SM100 comfort cutting mill (Retsch GmbH, Germany) after which they were analyzed for total N [29] and crude silica [53]. The plant samples were digested using the single dry ashing method [28] after which K, Ca, Mg, Cu, Zn, Fe, and Mn contents were determined using atomic absorption spectrophotometer (AAnalyst 800, Perkin Elmer Instruments, Norwalk, CT) whereas P was determined using Molybdenum Blue Method [32]. Nutrient uptake was calculated by multiplying nutrient content with plant dry weight. Rice plant nutrient recovery was calculated using the method of Dobermann [54].

### 2.7. Statistical Analysis

Analysis of variance (ANOVA) was used to detect significant differences among treatments whereas Tukey's test was used to compare treatment means using Statistical Analysis System version 9.2 [55].

## 3. Results and Discussion

**Figure 1:**
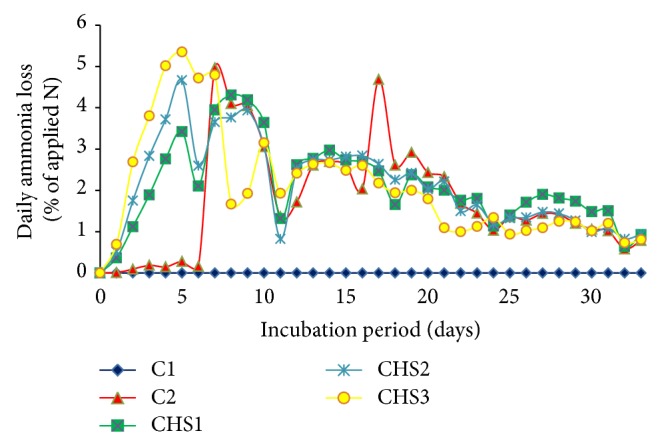
Daily ammonia loss for different treatments (C1: soil alone, C2: soil + complete fertilization, CHS1: soil + complete fertilization + 20 g rice straw compost + 2 g sawdust ash, CHS2: soil + complete fertilization + 40 g rice straw compost + 4 g sawdust ash, and CHS3: soil + complete fertilization + 60 g rice straw compost + 6 g sawdust ash) over 33 days of incubation.

Ammonia volatilization was observed for soil with complete fertilization without crude humic substances (C2) and soil with crude humic substances (CHS1, CHS2, and CHS3) on the first day of incubation (Figure 1). Ammonia loss from C2, CHS1, CHS2, and CHS3 lasted for 32 days. Ammonia volatilization decreased at 6, 11, 18, and 24 days of incubation and increased at 7, 12, 19, and 25 days of incubation in all the treatments except for C1. The fluctuation in ammonia volatilization during the incubation study was due to the reaction between urea and soil water to form NH_4_^+^ [24, 56]. As the soil surface rapidly dried due to air velocity in the chamber, NH_3_ from urea decreased at 6, 11, 18, and 24 days of incubation. Ammonia loss decreased when soil water was insufficient for the chemical reaction and it increased at 7, 12, 19, and 25 days of incubation upon addition of water. This observation is consistent with that of Bundan et al. [56] and Palanivell et al. [24]. No ammonia volatilization in soil alone (C1) suggests that soil alone did not contribute to ammonia loss. This observation is consistent with findings reported in previous studies [41, 56].

**Figure 2:**
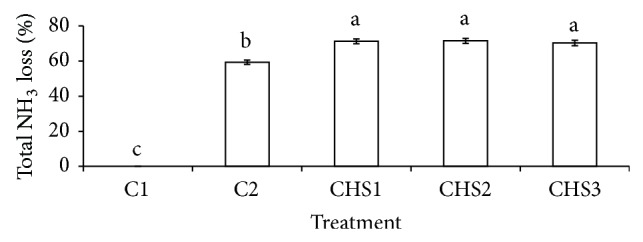
Total ammonia loss for different treatments (C1: soil alone, C2: soil + complete fertilization, CHS1: soil + complete fertilization + 20 g rice straw compost + 2 g sawdust ash, CHS2: soil + complete fertilization + 40 g rice straw compost + 4 g sawdust ash, and CHS3: soil + complete fertilization + 60 g rice straw compost + 6 g sawdust ash) at 33 days of incubation. Different alphabets indicate significant difference between means using Tukey's test at *P* ≤ 0.05. The error bars are the ± standard error of triplicates.

Treatments with crude humic substances (CHS1, CHS2, and CHS3) showed higher NH_3_ loss compared to the normal fertilization (C2) (Figure 2). The basic nature of the sawdust ash (pH 11.03) used in this study might have contributed to the NH_3_ volatilization in the treatments with crude humic substances (CHS1, CHS2, and CHS3). This is because rapid conversion of NH_4_^+^ ions to NH_3_ gas during urea hydrolysis occurs at higher pH [57, 58]. However, in a related study, application of crude humins from composts in nonwaterlogged condition showed no effect on NH_3_ volatilization compared with normal fertilization [23].

**Figure 3:**
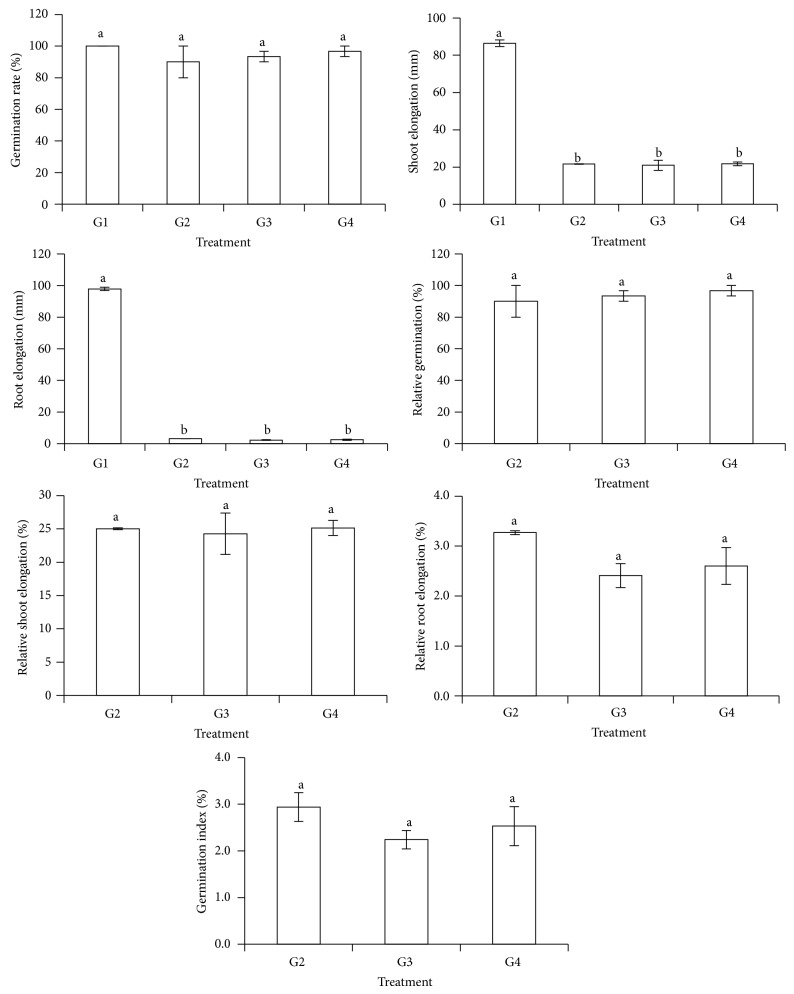
Treatments (G1: 100% sand, G2: 5% rice straw compost + 95% sand, G3: 10% rice straw compost + 80% sand, and G4: 15% rice straw compost + 85% sand) effect on rice seeds germination rate, shoot elongation, root elongation, relative germination, relative shoot elongation, relative root elongation, and germination index of rice seedlings at 7 days after seeding. Different alphabets indicate significant difference between means using Tukey's test at *P* ≤ 0.05. The error bars are the ± standard error of triplicates.

The effects of all the treatments on seed germination rate, relative germination, relative shoot elongation, relative root elongation, and germination index were statistically similar (Figure 3). Application of rice straw compost (G2, G3, and G4) showed no significant effects on seed germination rate and relative germination. However, rice straw compost (G2, G3, and G4) significantly reduced root elongation and shoot elongation compared with sand alone (G1) (Figure 3). Gallardo-Lara and Nogales [59] demonstrated that high N content in compost and compost rate can cause an inhibitory effect on seed germination and growth. Thus, this germination media test shows that the use of rice straw compost as a germination medium for rice seeds is not suitable. This is further supported by the lower germination index (less than 80%) of the rice seeds on G2, G3, and G4 [50]. In a related study, rice straw was transformed into soil-like substrate through aerobic fermentation and bioconversion using fungi and worms before using the product as a planting medium [60]. Hence, sand alone (G1) was used as germination medium for the rice seedlings production in our pot study.

**Figure 4:**
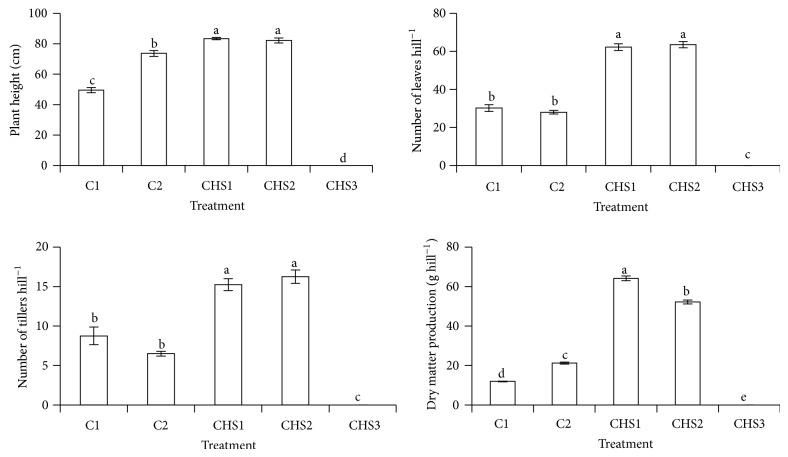
Treatments (C1: soil alone, C2: soil + complete fertilization, CHS1: soil + complete fertilization + 20 g rice straw compost + 2 g sawdust ash, CHS2: soil + complete fertilization + 40 g rice straw compost + 4 g sawdust ash, and CHS3: soil + complete fertilization + 60 g rice straw compost + 6 g sawdust ash) effect on plant height, number of leaves, tillers, and dry matter production of rice plant at 90 days after seeding. Different alphabets indicate significant difference between means using Tukey's test at *P* ≤ 0.05. The error bars are the ± standard error of quadruplicates.

**Figure 5:**
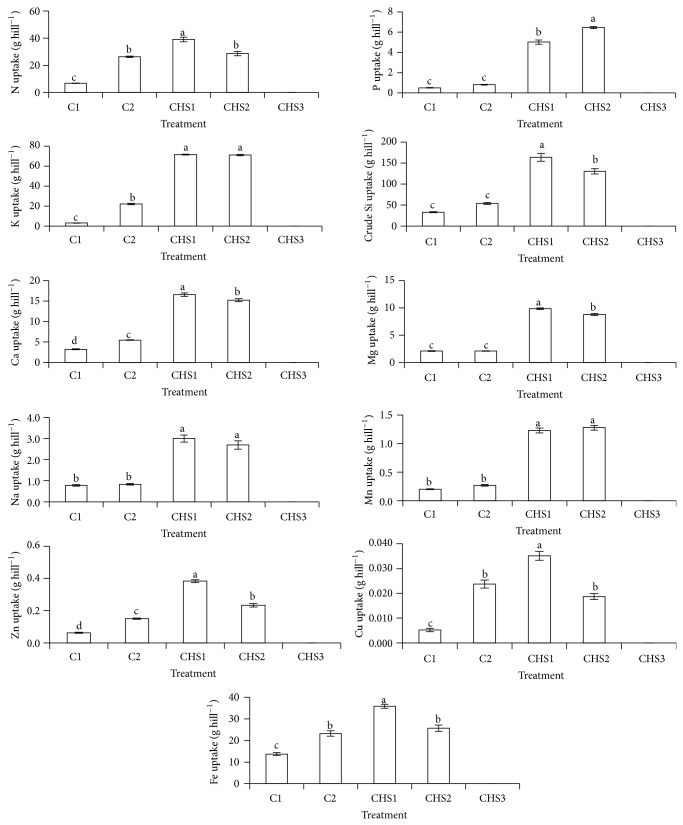
Treatments (C1: soil alone, C2: soil + complete fertilization, CHS1: soil + complete fertilization + 20 g rice straw compost + 2 g sawdust ash, CHS2: soil + complete fertilization + 40 g rice straw compost + 4 g sawdust ash, and CHS3: soil + complete fertilization + 60 g rice straw compost + 6 g sawdust ash) effect on N, P, K, crude silica, Ca, Mg, Na, Cu, Zn, Mn, and Fe uptake of rice plant at 90 days after seeding. Different alphabets indicate significant difference between means using Tukey's test at *P* ≤ 0.05. The error bars are the ± standard error of quadruplicates.

**Figure 6:**
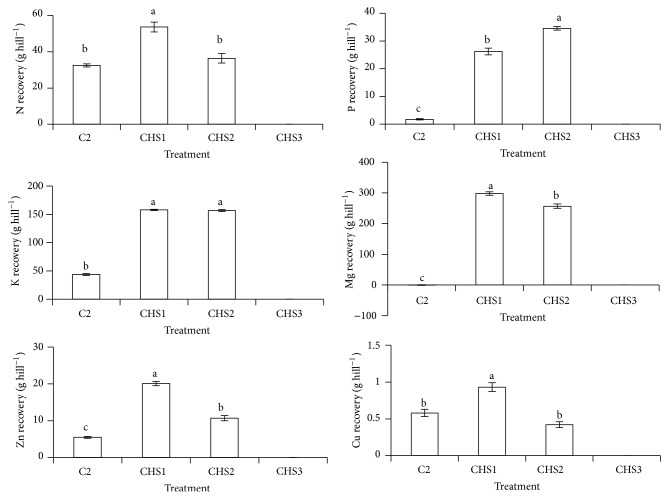
Treatments (C1: soil alone, C2: soil + complete fertilization, CHS1: soil + complete fertilization + 20 g rice straw compost + 2 g sawdust ash, CHS2: soil + complete fertilization + 40 g rice straw compost + 4 g sawdust ash, and CHS3: soil + complete fertilization + 60 g rice straw compost + 6 g sawdust ash) effect on rice plant N, P, K, Mg, Cu, and Zn recovery at 90 days after seeding. Different alphabets indicate significant difference between means using Tukey's test at *P* ≤ 0.05. The error bars are the ± standard error of quadruplicates.

CHS1 and CHS2 significantly improved rice plant height, dry matter production, number of leaves, and tillers compared with normal fertilization (C2) (Figure 4). The lowest crude humic substances rate (CHS1) showed the highest dry matter production compared with other treatments. According to Smith and Dilday [61], high nutrients uptake (especially N and silica) increased rice plant growth and dry matter production, and this observation is consistent with the effect of CHS1 as this treatment showed the highest nutrient uptake (N, crude Si, Ca, Mg, Zn, Cu, and Fe) and recovery (N, Mg, Zn, and Cu) compared with other treatments (Figures 5 and 6). The rice plants under the highest crude humic substances rate (CHS3) died (Figures 4, 5, and 6). The high soil pH (pH 7.13) of CHS3 could be the reason for the rice plant death, because the rice plant variety of MR219 grows well within soil pH of 5.5 to 6.5 [42].

The treatments with crude humic substances (CHS1 and CHS2) showed higher nutrients uptake (K, P, crude Si, Ca, Mg, Na, Mn, and Zn) and recovery (P, K, and Mg) (Figures 5 and 6) compared with normal fertilization (C2). The rice plants with higher crude humic substances (CHS2) showed the highest P uptake and recovery compared with other treatments (Figures 5 and 6) whereas the lowest crude humic substances rate (CHS1) showed the highest uptake of N, crude Si, Ca, Mg, Zn, Cu, and Fe and recovery of N, Mg, Zn, and Cu compared with other treatments (Figures 5 and 6). The highest nutrient uptake and recovery in CHS1 was because of the highest dry matter production in CHS1 (Figure 4).

**Figure 7:**
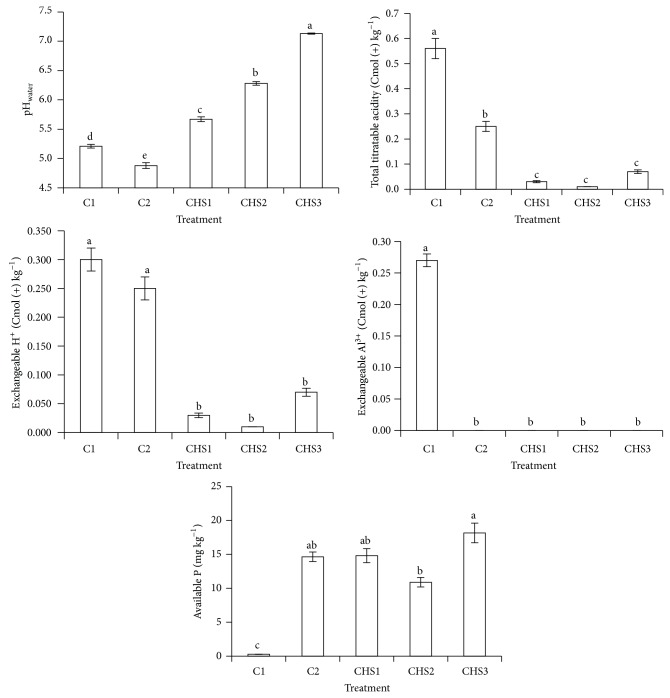
Treatments (C1: soil alone, C2: soil + complete fertilization, CHS1: soil + complete fertilization + 20 g rice straw compost + 2 g sawdust ash, CHS2: soil + complete fertilization + 40 g rice straw compost + 4 g sawdust ash, and CHS3: soil + complete fertilization + 60 g rice straw compost + 6 g sawdust ash) effect on soil pH, total titratable acidity, exchangeable H^+^, exchangeable Al^3+^, and available P content at 90 days after seeding. Different alphabets indicate significant difference between means using Tukey's test at *P* ≤ 0.05. The error bars are the ± standard error of quadruplicates.

The treatments with the different rates of crude humic substances (CHS1, CHS2, and CHS3) significantly increased soil pH compared with C1 (soil alone) and C2 (normal fertilization) (Figure 7). Soil pH also increased with increasing rate of crude humic substances (CHS1 < CHS2 < CHS3) (Figure 7) due to the high pH and basic cations of the sawdust ash used in this study (Table 2). Soil total titratable acidity and exchangeable H^+^ were significantly lower in the crude humic substances treatments (CHS1, CHS2, and CHS3) compared with soil alone (C1) and normal fertilization (C2) (Figure 7). The high pH and basic cations of the sawdust ash (Table 2) in CHS1, CHS2, and CHS3 might have reduced soil titratable acidity and exchangeable H^+^. Soil alone treatment (C1) showed the highest total titratable acidity and exchangeable Al^3+^ compared with other treatments because some of the P fertilizer (ERP) in C2, CHS1, CHS2, and CHS3 might have reduced exchangeable Al^3+^ and total titratable acidity compared with soil alone (C1) as in acidic soils, hydroxides and oxides of Al^3+^ are precipitated by H_2_PO_4_^−^ as variscite and various minerals of the plumbogummite group [14]. Available P in normal fertilization and crude humic substances (CHS1, CHS2, and CHS3) were statistically similar (Figure 7). However, the highest rate of crude humic substances (CHS3) showed higher available P compared with CHS2. The lower available P in CHS2 compared with CHS3 was due to the higher P uptake and recovery by rice plants under CHS2 as those under CHS3 died.

**Figure 8:**
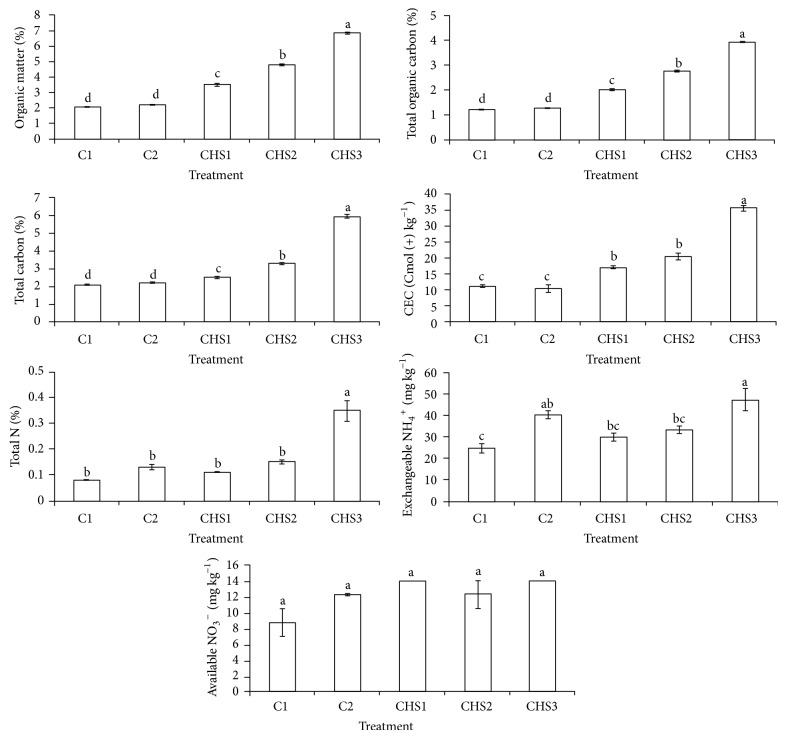
Treatments (C1: soil alone, C2: soil + complete fertilization, CHS1: soil + complete fertilization + 20 g rice straw compost + 2 g sawdust ash, CHS2: soil + complete fertilization + 40 g rice straw compost + 4 g sawdust ash, and CHS3: soil + complete fertilization + 60 g rice straw compost + 6 g sawdust ash) effect on soil total carbon, total organic carbon, organic matter, cation exchange capacity, exchangeable NH_4_^+^, available NO_3_^−^, and total nitrogen content at 90 days after seeding. Different alphabets indicate significant difference between means using Tukey's test at *P* ≤ 0.05. The error bars are the ± standard error of quadruplicates.

CHS1, CHS2, and CHS3 increased soil organic matter (OM), total organic carbon (TOC), total carbon (TC), and cation exchange capacity (CEC) compared with soil alone (C1) and normal fertilization (C2) (Figure 8). Higher OM, TOC, TC, and CEC of the crude humic substances applied soil (CHS1, CHS2, and CHS3) were because of the inherent contents of OM, TOC, TC, and higher CEC of rice straw compost (Table 2). The highest rate of crude humic substances (CHS3) showed the highest soil total N compared to other treatments and this was due to no N depletion in soil because the rice plants could not survive. Normal fertilization (C2) and crude humic substances treatments (CHS1, CHS2, and CHS3) showed similar effect on exchangeable NH_4_^+^ and available NO_3_^−^. The higher NH_3_ volatilization regardless of treatment resulted in lower contents of exchangeable NH_4_^+^ and available NO_3_^−^ in soil (Figure 2).

**Figure 9:**
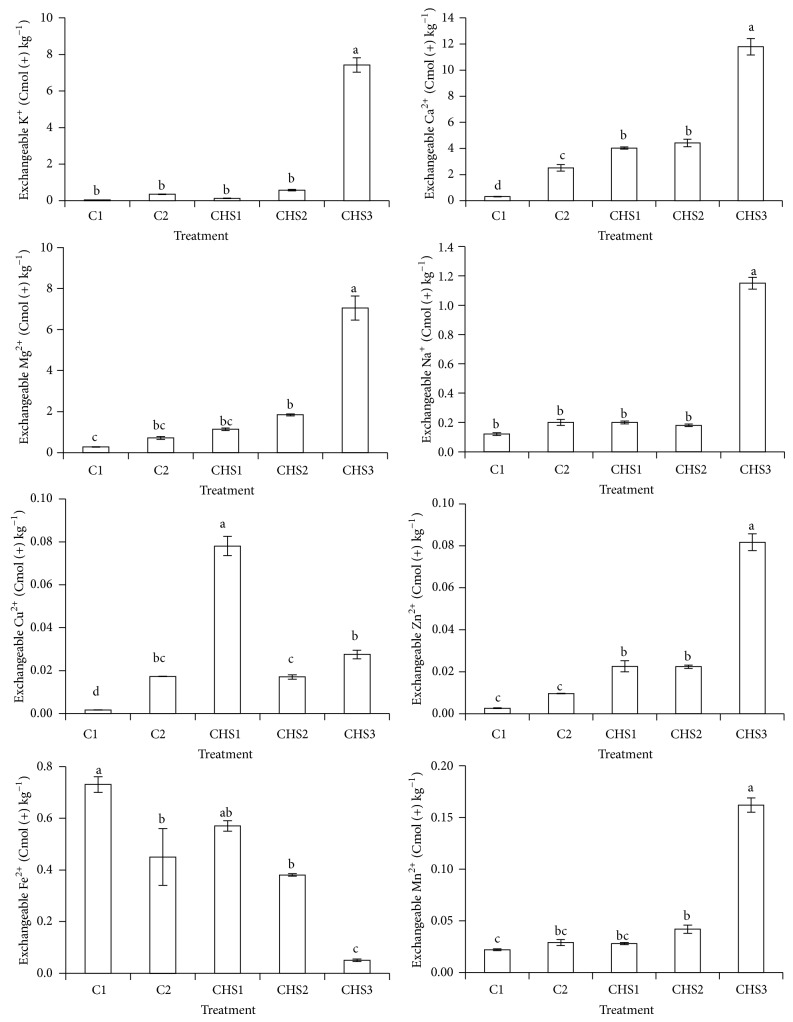
Treatments (C1: soil alone, C2: soil + complete fertilization, CHS1: soil + complete fertilization + 20 g rice straw compost + 2 g sawdust ash, CHS2: soil + complete fertilization + 40 g rice straw compost + 4 g sawdust ash, and CHS3: soil + complete fertilization + 60 g rice straw compost + 6 g sawdust ash) effect on selected soil exchangeable cations (K^+^, Ca^2+^, Mg^2+^, Na^+^, Cu^2+^, Zn^2+^, Fe^2+^, and Mn^2+^) content at 90 days after seeding. Different alphabets indicate significant difference between means using Tukey's test at *P* ≤ 0.05. The error bars are the ± standard error of quadruplicates.

The effects of the different treatments on selected exchangeable cations at 90 days of seeding are shown in Figure 9. The treatment with the highest rate of crude humic substances (CHS3) showed the highest soil exchangeable K^+^, Ca^2+^, Mg^2+^, Na^+^, Zn^2+^, and Mn^2+^ compared with other treatments due to less nutrients depletion in the soil as the rice plants died during this study. CHS1, CHS2, and CHS3 increased exchangeable Ca^2+^ and Zn^2+^ compared to normal fertilization (C2). The treatment with the lowest crude humic substances (CHS1) showed the highest exchangeable Cu^2+^ compared with other treatments because of antagonistic effect of Cu and Zn [62]. Soil exchangeable Fe^2+^ (CHS1 > CHS2 > CHS3) decreased with increasing rate of crude humic substances (CHS1 < CHS2 < CHS3) due to increase in soil pH (Figure 7) [63].

In this present study, the lowest crude humic substance rate (CHS1) is considered optimum rate for increasing MR219 variety rice plants' dry matter production, uptake of N, crude Si, Ca, Mg, Zn, Cu, and Fe, and recovery of N, Mg, Zn, and Cu. However, application of crude humic substances at the rate of 40 g pot^−1^ (CHS2) showed similar effect as that of CHS1 on plant height, number of leaves, tillers, uptake of K, P, crude Si, Ca, Mg, Na, Mn, and Zn, and recovery of P, K, and Mg.

## 4. Conclusions

Amending an acid soil (Typic Paleudults) with crude humic substances in waterlogged condition increased ammonia loss. However, application of the lowest crude humic substances rate (20 g pot^−1^) improved rice plant dry matter production, nutrients uptake, recovery, and soil chemical properties. Therefore, crude humic substances could be used to amend acid soils in flooded rice fields to improve MR219 rice plant growth and soil chemical properties but long term field evaluation is essential to consolidate the findings of this study. This aspect is being embarked on in our ongoing field experiments.

## Figures and Tables

**Table 1:** Selected chemical properties of Typic Paleudults (Bekenu Series) soil before incubation and pot study.

Property	Current study	Standard data range^*^ (0–36 cm)
pH_water_	4.41	4.6–4.9
Bulk density (g cm^−3^)	1.16	NA
TOC (%)	1.43	0.57–2.51
Total N (%)	0.08	0.04–0.17
Exchangeable NH_4_^+^ (mg kg^−1^)	21.02	NA
Available NO_3_^−^ (mg kg^−1^)	7.01	NA
Available P (mg kg^−1^)	4.85	NA

(Cmol (+) kg^−1^)		
CEC	11.97	3.86–8.46
Exchangeable K^+^	0.10	0.05–0.19
Exchangeable Ca^2+^	0.25	NA
Exchangeable Mg^2+^	0.34	NA
Exchangeable Na^+^	0.22	NA
Exchangeable Fe^2+^	0.19	NA
Exchangeable Cu^2+^	Trace	NA
Exchangeable Zn^2+^	0.01	NA
Exchangeable Mn^2+^	0.02	NA
Total titratable acidity	0.86	NA
Exchangeable H^+^	0.64	NA
Exchangeable Al^3+^	0.22	NA

NA: not available; ^*^Paramanathan [34].

**Table 2:** Selected chemical properties of rice straw compost and sawdust ash.

Property	Rice straw compost	Sawdust ash
pH	7.58	11.03
CEC (Cmol (+) kg^−1^)	93.60	nd
Molarity (w : v = 1 : 100) (M)	nd	0.002

(%)		
Organic matter	74.00	nd
Total organic carbon	42.92	nd
Humic acids	10.97	nd
Crude humins	87.93	nd
Total N	1.85	nd
Total P	0.75	0.47
Total K	2.42	0.27
Total Ca	1.20	7.63
Total Mg	0.75	1.09
Total Na	0.24	0.04
Total Fe	0.25	3.57
Total Zn (mg kg^−1^)	164	683
Total Mn (mg kg^−1^)	223	1833
Total Cu (mg kg^−1^)	71	213

**Table 3:** Fertilization schedule for the pot study.

Days after seeding (DAS)	Urea	ERP	MOP	Kieserite	Zncobor
	(g pot^−1^)				
15 DAS	0.55	0.79	0.24	—	—
35 DAS	0.40	—	0.24	0.06	0.53
50 and 70 DAS	0.18	0.30	0.20	0.05	—
